# COVID-19 (Coronavirus) Pandemic: Information Sources Channels for the Public Health Awareness

**DOI:** 10.1177/1010539520927261

**Published:** 2020-05-19

**Authors:** Muhammad Yousuf Ali, Rubina Bhatti

**Affiliations:** 1 The Aga Khan University, Karachi, Sindh, Pakistan; 2 Department of Library and Information Science, The Islamia University, Bahawalpur, Punjab, Pakistan

**Keywords:** COVID-19, coronavirus, public health, librarian, health information, pandemic

## Abstract

The main purpose of this paper is to highlight the important information sources of the Public Health awareness used by the library and information sources in this Pandemic situation. Social distancing phase Information professional used a different medium to connect with their patron and try to serve the best manner. The role of the information professional in health information and health literacy is very vital. Information professional public health awareness information with the library patrons and the general public. In this paper, the researchers provide a brief introduction to different information channel support in information dissemination.

## Introduction

COVID-19 public health awareness is the most effective tool to protect this crisis. Public health awareness helps and reduces the intensity of spreading rate and reduces death rate, and precautionary measures are required to control this pandemic disease. To combat this pandemic condition, the roles of a librarian and information professional are very vital in three dimensions: public health awareness for prevention measures; support to research team/researchers and faculty about the latest developments and research and literature; and service to regular library users and/or information seekers.^1^ The authors explore how information about public health is sought and used in this emergency/lockdown situation.

## Information Channel

Following information, channels are used by the librarians and information professionals during the pandemic of COVID-19 to facilitate public health awerness.

### Mobile Apps

COVID-19 (coronavirus) is a contagious disease. Mobile apps are used to educate the people to know about the early-stage diagnosis symptoms of COVID-19 and to inform the general public about the disease. Health organization, IT companies, and universities worldwide have introduced mobile applications, and this will reduce the influx load to the hospital/health care center.

### Artificial Intelligence–Based Chatbots

Artificial intelligence–based chatbots are also one the successful tools used to chat with the general public. This chatbot is designed in different local and international languages by developers, and one can chat 24/7 and get information about coronavirus symptoms, diagnosis, and precautionary measures.

### Social Media Trolling

Social media platforms are also one the fastest mode/medium of public health awareness, and twitter # tag information provided^2^ about what going on all over the world in the fastest mode. Facebook, WhatsApp, and Instagram are also other renowned forums of message sharing to the public about the latest updates of the situation. Patient and their attendant also engage via social media and share their experience to create awareness to the public.

In addition to authentic information, some fake news and information are also shared via social media about this pandemic. Such types of information create panic in public health. Social media or alternative news create some fear and rumors about the pandemic during the lockdown period.^3,4^

### Video-Based Lecture

Video-based lectures on YouTube, Vimeo, and Dailymotion are other sources where infectious disease experts share video clips about coronavirus symptoms, cure, and possible measure to avoid this pandemic.

### Electronic Resources

Medical staff, faculty members, researchers, health support organizations, and paramedical staff support disseminating the latest developments regarding the vaccination, diagnosis kits, and latest literature published on the topic. All the renowned databases provide free access to COVID-19 coronavirus literature. Renowned and leading publishers, that is, Elsevier, Oxford, Wiley, BMJ, Nature, Sage, Emerald, Cambridge, and others, provide free access to the latest literature on coronavirus in the fight against coronavirus.

## Conclusion

In this COVID-19 pandemic, social distance is one of the keys to protecting ourselves. In this information age, public health awareness is key to minimize causalities, and librarian and information professional can play a vital role to disseminate the information with health care workers, society, and communities. maintaining social distance is important during the lockdown phase. These information channels play a vital role in informing and updating public health information to the general public and health care professionals.
